# Draft Genome Assembly of the Poultry Red Mite, *Dermanyssus gallinae*

**DOI:** 10.1128/MRA.01221-18

**Published:** 2018-11-08

**Authors:** Stewart T. G. Burgess, Kathryn Bartley, Francesca Nunn, Harry W. Wright, Margaret Hughes, Matthew Gemmell, Sam Haldenby, Steve Paterson, Stephane Rombauts, Fiona M. Tomley, Damer P. Blake, James Pritchard, Sabine Schicht, Christina Strube, Øivind Øines, Thomas Van Leeuwen, Yves Van de Peer, Alasdair J. Nisbet

**Affiliations:** a Moredun Research Institute (MRI), Edinburgh, United Kingdom; b Centre for Genomic Research, Institute of Integrative Biology, University of Liverpool, Liverpool, United Kingdom; c Department of Plant Biotechnology and Bioinformatics, Ghent University, Ghent, Belgium; d VIB Center for Plant Systems Biology, Ghent, Belgium; e Bioinformatics Institute Ghent, Ghent University, Ghent, Belgium; f Department of Pathology and Population Sciences, Royal Veterinary College, Hatfield, Hertfordshire, United Kingdom; g Institute for Parasitology, Centre for Infection Medicine, University of Veterinary Medicine Hannover, Hannover, Germany; h Norwegian Veterinary Institute, Oslo, Norway; i Department of Plants and Crops, Ghent University, Ghent, Belgium; j Department of Biochemistry, Genetics, and Microbiology, University of Pretoria, Pretoria, South Africa; University of Maryland School of Medicine

## Abstract

The poultry red mite, *Dermanyssus gallinae*, is a major worldwide concern in the egg-laying industry. Here, we report the first draft genome assembly and gene prediction of *Dermanyssus gallinae*, based on combined PacBio and MinION long-read *de novo* sequencing. The ∼959-Mb genome is predicted to encode 14,608 protein-coding genes.

## Abstract (precis)

The poultry red mite, *Dermanyssus gallinae*, is a major worldwide concern in the egg-laying industry. Here, we report the first draft genome assembly and gene prediction of *Dermanyssus gallinae*, based on combined PacBio and MinION long-read *de novo* sequencing.

## ANNOUNCEMENT

Infestation of hen houses with the poultry red mite, *Dermanyssus gallinae*, causes major health and welfare concerns for the egg-producing industry worldwide (1, 2), costing the European Union poultry industry >€231 million annually (see https://www.pluimveeweb.nl/artikelen/2017/01/schade-bloedluis-21-miljoen-euro/). Control relies on the treatment of premises with acaricide sprays or systemic treatments with isoxazoline-based therapeutics (2, 3). Concerns over residues, environmental contamination, and acaricide resistance threaten sustainability and have highlighted interest in developing alternative control methods (1). These novel approaches require comprehensive genomic information and genome-based tools for gene expression analysis and trait mapping.

Adult female *D. gallinae* mites were harvested from a commercial poultry shed in Scotland, and freshly laid mite eggs were collected over 24 h. Contaminating material was removed by washing in 0.1% benzalkonium chloride before rinsing in double-distilled water (ddH_2_O). Approximately 900 µl of eggs were gently homogenized in 12 ml of SDS/RNase A/proteinase K buffer, and genomic DNA (gDNA) was extracted using the SDS-proteinase K method (4). DNA integrity was assessed by gel electrophoresis and quantified using a Qubit double-stranded DNA (dsDNA) broad-range (BR) kit. PacBio sequencing libraries were generated from high-molecular-weight gDNA using the PacBio SMRTbell template prep kit v1.0 according to the manufacturer’s instructions and sequenced using 10 single-molecule real-time (SMRT) cells on a PacBio RS II instrument. Sequences were assembled using Canu v1.6 (5) with an estimated genome size of 500 Mb. The resulting assembly was scaffolded with low-coverage Oxford Nanopore Technologies MinION reads (6 gigabases [Gb] of sequence data generated with the 1D ligation kit on an R9.4 flow cell) using PBJelly 2 (6) followed by 8 iterations of genome polishing with Arrow (7). The final assembly contained 7,171 contigs with an *N*_50_ value of 278,630 bp and an *L*_50_ value of 800 contigs, the largest scaffold having 3,781,415 bp and an overall genome GC content of 44.6%. The assembled genome size was 959 Mb, and 63.5 Gb of PacBio sequencing data provided ∼66× coverage.

Gene prediction employed the MAKER pipeline v2.31.8 (8) with Semi-HMM-based Nucleic Acid Parser (SNAP) *ab initio* gene predictions (9) using proteins from Metaseiulus occidentalis, Ixodes scapularis, and the UniProt and Swiss-Prot protein databases to support gene models. Full-length transcripts were obtained from the total RNA from mixed-stage *D. gallinae* and were analyzed with PacBio Iso-Seq pipelines within the PacBio SMRT Portal v5.0.1.10424 (7) (minimum Quiver/Arrow accuracy, 0.85; minimum GMAP alignment identity, 0.8), generating 13,612 high-quality and 53,082 low-quality isoforms post-Quiver polishing (mean read length of 1,142 bp). Repeat sequences were detected using RepeatModeler (http://www.repeatmasker.org) and provided to MAKER for repeat masking. This identified 14,608 predicted protein-coding genes with an average length of 1,294 bp. The core eukaryotic protein-coding gene presence was assessed with BUSCO (10) v2.0 (Arthropoda set) with 93% of the single-copy orthologs present (73% single copies, 13% duplicates, 7% fragments). Predicted proteins were annotated with Pfam information using InterProScan v5.22-61.0 (11). Those containing a Pfam domain or an annotation score (AED) of <1 were accepted in the final output of 14,608 genes/transcripts. BLAST hits against the NCBI nonredundant (nr) database (July 2018) were identified for 13,840 genes, and Gene Ontology (GO), performed in Blast2GO (10), resulted in the assignment of GO terms for 11,624 genes and functional annotation of 10,914 genes. Unless otherwise stated above, default algorithm parameters were used.

### Data availability.

This whole-genome shotgun project has been deposited at DDBJ/ENA/GenBank under the accession number QVRM00000000. The version described in this paper is version QVRM01000000. The mixed-stage *D. gallinae* PacBio Iso-Seq data have been deposited at the NCBI Sequence Read Archive under the accession number PRJNA494800.
